# Effectiveness and safety of coronavirus disease 2019 vaccines

**DOI:** 10.1097/MCP.0000000000000948

**Published:** 2023-02-24

**Authors:** Ting Shi, Chris Robertson, Aziz Sheikh

**Affiliations:** a Usher Institute, Edinburgh Medical School, University of Edinburgh, Edinburgh; b Department of Mathematics and Statistics, University of Strathclyde, Glasgow; c Public Health Scotland, Glasgow, Scotland, UK

**Keywords:** coronavirus disease 2019, severe acute respiratory syndrome coronavirus 2, vaccine, vaccine effectiveness, vaccine safety

## Abstract

To review and summarise recent evidence on the effectiveness of coronavirus disease 2019 (COVID-19) vaccines against severe acute respiratory syndrome coronavirus 2 (SARS-CoV-2) infection and COVID-19 hospitalisation and death in adults as well as in specific population groups, namely pregnant women, and children and adolescents. We also sought to summarise evidence on vaccine safety in relation to cardiovascular and neurological complications. In order to do so, we drew primarily on evidence from two our own data platforms and supplement these with insights from related large population-based studies and systematic reviews.

**Recent findings:**

All studies showed high vaccine effectiveness against confirmed SARS-CoV-2 infection and in particular against COVID-19 hospitalisation and death. However, vaccine effectiveness against symptomatic COVID-19 infection waned over time. These studies also found that booster vaccines would be needed to maintain high vaccine effectiveness against severe COVID-19 outcomes. Rare cardiovascular and neurological complications have been reported in association with COVID-19 vaccines.

**Summary:**

The findings from this paper support current recommendations that vaccination remains the safest way for adults, pregnant women, children and adolescents to be protected against COVID-19. There is a need to continue to monitor the effectiveness and safety of COVID-19 vaccines as these continue to be deployed in the evolving pandemic.

## INTRODUCTION

Coronavirus disease 2019 (COVID-19) vaccination programmes have been rolled out globally as the key strategy to control and minimise the impact of the COVID-19 pandemic. Three vaccines have mainly been used in the UK, namely BNT162b2 (Pfizer-BioNTech), ChAdOx1 nCoV-19 (Oxford-AstraZeneca) and mRNA-1273 (Moderna). There are many studies reporting safety and effectiveness of the COVID-19 vaccines against severe acute respiratory syndrome coronavirus 2 (SARS-CoV-2) infection and severe COVID-19 outcomes [1^▪^]. In this review, we summarise recent evidence around COVID-19 vaccine effectiveness against confirmed SARS-CoV-2 infection and COVID-19 hospitalisation and death in adults as well as the vaccine effectiveness in some specific population groups, i.e., pregnant women, children and adolescents. We also aim to summarise recent evidence on vaccine safety regarding cardiovascular and neurological complications.

In doing so, we draw primarily on evidence from two our own data platforms (i.e., Early Pandemic Evaluation and Enhanced Surveillance of COVID-19 (EAVE II) and Data and Connectivity: COVID-19 Vaccines Pharmacovigilance (DaC-VaP)) and supplement these with insights from related large population-based studies and systematic reviews. EAVE II is a Scotland-wide COVID-19 surveillance platform that has been used to track and forecast the epidemiology of COVID-19, inform risk stratification assessment, and investigate vaccine effectiveness and safety [2]. It comprises national healthcare datasets on 5.4 million people (approximately 99% of the Scottish population) deterministically linked through the Community Health Index number, which is a unique identifier used for all health-care contact across Scotland. DaC-VaP is a UK-wide collaboration looking at the safety and effectiveness of COVID-19 vaccines using linked electronic health record data in all four UK nations [3]. 

**Box 1:**
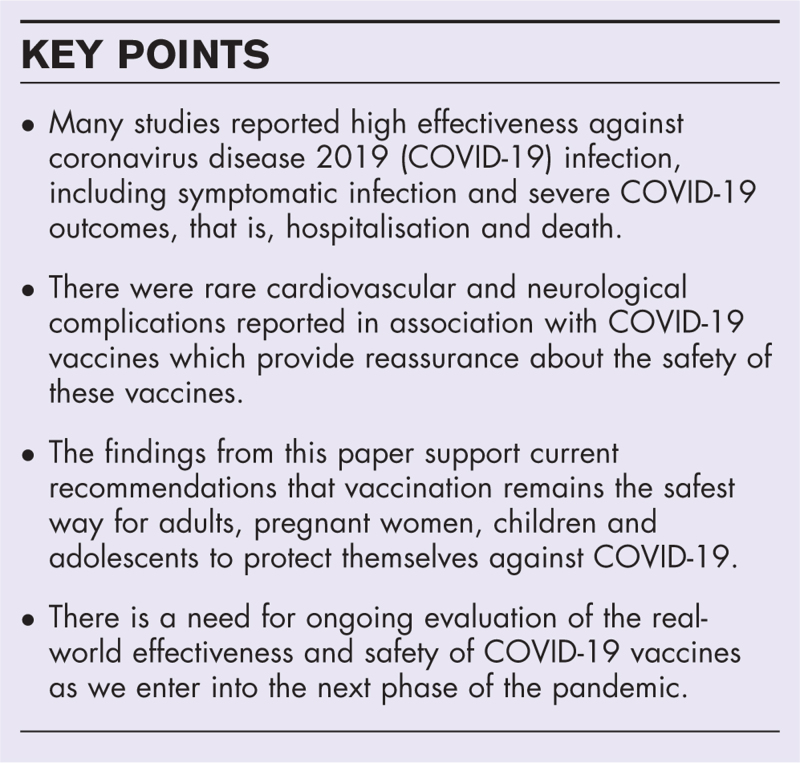
no caption available

## CORONAVIRUS DISEASE 2019 VACCINE EFFECTIVENESS

### Coronavirus disease 2019 vaccine effectiveness in adults

In early 2021, we conducted a population-based national prospective cohort study using the EAVE II platform. This study comprised linked vaccination, primary care, real-time reverse transcription polymerase chain reaction (RT-PCR) testing, and hospital admission patient records for 5.4 million people in Scotland registered at 940 general practices. The study found that mass roll-out of the first doses of the BNT162b2 and ChAdOx1 nCoV-19 vaccines were associated with substantial reductions in the risk of COVID-19 hospital admission among adults in Scotland [4^▪▪^]. Between December 8, 2020 and February 22, 2021, the first dose of the BNT162b2 vaccine had a vaccine effectiveness (VE) of 91% [95% confidence interval (CI) 85–94] in reducing COVID-19 related hospital admission at 28–34 days post-COVID-19 vaccination among adults. VE for the ChAdOx1 nCoV-19 at 28–34 days post-COVID-19 vaccination was 88% (95% CI 75–94). The combined VE against COVID-19 related hospital admission was 89% (95% CI 83–92) at the same time interval. When we restricted the analysis to those aged at least 80 years old, the combined VE against COVID-19 hospital admission was 83% (95% CI 72–89) at 28–34 days post COVID-19 vaccination. This first national analysis provided considerable reassurance based on real-world evidence that vaccines were highly effective in reducing the risk of serious COVID-19 outcomes and that they also provided very high levels of protection in the elderly.

The emergence of variants of concern (VOCs) has raised important questions about VE. Using the EAVE II platform, we have been able to show that VE against serious COVID-19 outcomes has remained high in fully vaccinated individuals infected with the Delta and Omicron VOCs and their key sub-lineages [5–8,9^▪▪^]. Specifically, our first national VE study mentioned above was against Alpha VOC infection when the vaccines were trialled against wild type [4^▪▪^]. The VE against death from the Delta VOC at least 14 days after the second vaccine dose was 90% (95% CI 83–94) for BNT162b2 and 91% (95% CI 86–94) for ChAdOx1 nCoV-19;[9^▪▪^] VE against symptomatic SARS-CoV-2 infection due to AY.4.2 (Delta plus – a sub-lineage of the Delta VOC) was 73% (95% CI 62–81) post 14 days of second vaccine dose [7]. The protection was consistent across BNT162b2, ChAdOx1 nCoV-19 and mRNA-1273. Both BNT162b2 and ChAdOx1 nCoV-19 vaccines were effective in reducing the risk of SARS-CoV-2 infection and COVID-19 hospitalisation in people with the Delta VOC, but these effects on infection appeared to be diminished when compared to those with the Alpha VOC [8].

VE of COVID-19 vaccination against confirmed SARS-CoV-2 infection and COVID-19 hospitalisation or death wanes over time [10]. Investigating vaccine waning was complicated by the emergence of new variants (specifically the Delta variant of concern in the UK). To investigate potential waning, our Scottish EAVE II partnered with colleagues in Brazil (where the Gamma variant of concern had emerged) in 2021. In Scotland, VE of two-dose ChAdOx1 nCoV-19 vaccine against COVID-19 related hospital admission and death in adults was 83.7% (95% CI 79.7–87.0) at 2–3 weeks, 75.9% (95% CI 72.9–78.6) at 14–15 weeks and 63.7% (95% CI 59.6–67.4) at 18–19 weeks; while in Brazil, the pattern of results was similar with VE reducing from 86.4% (95% CI 85.4–87.3) to 59.7% (95% CI 54.6–64.2) and 42.2% (95% CI 32.4–50.6) at the corresponding time points, respectively [11^▪▪^]. From this we concluded that vaccine waning was occurring against severe COVID-19 outcomes underscoring the need for booster vaccines. A systematic review and meta-regression analysis of 18 studies found that, on average, VE against severe COVID-19 disease remained high albeit decreasing by 10.0% (95% CI 6.1–15.4) from 1 month to 6 months after full vaccination in people of all ages [12^▪▪^]. There was in contrast a more substantial 20–30% reduction in protection against infection and milder disease in the six months following full vaccination [12^▪▪^].

There is a growing body of evidence that booster doses enhance protection. EAVE II study has reported that booster vaccines were effective in preventing symptomatic infection with a 57% (54–60) reduction in the risk of symptomatic Omicron VOC in comparison to individuals who were positive with SARS-CoV-2 at least 25 weeks after the second vaccine dose [6]. A team from Brazil found that BNT162b2 booster vaccine 6 months after completion of the primary vaccination schedule increased VE against confirmed SARS-CoV-2 infection from 34.7% (95% CI 33.1–36.2) at least 180 days after second dose of CoronaVac (Sinovac Biotech) to 92.7% (95% CI 91.0–94.0) and against severe outcomes (hospitalisation or death) from 72.5% (95% CI 70.9–74.0) to 97.3% (95% CI 96.1–98.1) 14–30 days after the booster dose [13].

Our recent UK-wide analysis of risk of serious COVID-19 outcomes following the first booster using the DaC-VaP platform has identified specific sub-populations who remain at high risk of serious COVID-19 outcomes who have then been prioritised for second dose boosters and COVID-19 therapeutics: older adults aged at least 80 years [adjusted rate ratio (aRR) 3.60 (95% CI 3.45–3.75)], those with five or more comorbidities [aRR 9.51 (95% CI 9.07–9.97)], being male [aRR 1.23 (95% CI 1.20–1.26)], and individuals with certain underlying health conditions, i.e., those receiving immunosuppressants [aRR 5.80 (95% CI 5.53–6.09)], and those with stage 5 chronic kidney disease [aRR 3.71 (95% CI 2.90–4.74)] [14^▪^].

### Coronavirus disease 2019 vaccine effectiveness in pregnant women

The EAVE II platform has been used to create a sub-cohort of pregnant women - COVID-19 in Pregnancy in Scotland (COPS) cohort. This cohort is one of the national cohorts of pregnant women that includes not only women who give births but also women who are pregnant and who subsequently do not give births either because of a termination or miscarriage. Our analysis of Scotland-wide data found that vaccine uptake was much lower in pregnant women than in the general female population aged 18–44 years: our analysis was undertaken in October 2021 and found, 32.3% of women who gave birth received two doses of vaccine compared to 77.4% in all women in the same age group irrespective of pregnancy [15^▪▪^]. A subsequent analysis of this pregnancy cohort has found that mothers who had received two or more doses of vaccination are likely protected against neonatal SARS-CoV-2 infection (all 12 cases of neonatal SARS-CoV-2 infection occurred in women who had not received at least two doses of vaccine when they had SARS-CoV-2 infection during pregnancy) [16].

A systematic review of 10 observational studies found that COVID-19 vaccination during pregnancy was associated with a reduced number of SARS-CoV-2 infections [odds ratio (OR) 0.56 (95% CI 0.47–0.67)] and COVID-19 related hospitalisations [OR 0.50, 95% CI (0.31–0.82)] [17]. On the other hand, COVID-19 vaccination was not associated with adverse outcomes in pregnancy, such as preeclampsia, eclampsia, stroke, postpartum haemorrhage, miscarriage prior to 20 weeks’ gestation or ectopic pregnancy [17,18^▪^]. Furthermore, the COVID-19 vaccine was associated with a decrease in neonatal COVID-19 intensive care unit (ICU) admission [OR 0.85 (95% CI 0.81–0.90)].

### Coronavirus disease 2019 vaccine effectiveness in children and adolescents

In a recent analysis involving a partnership between the EAVE II team and colleagues in Brazil, we found that VE against symptomatic SARS-CoV-2 infection was reported highest at 14–27 days post second dose among adolescents aged 12–17 years [19]. VE was lower during the Omicron dominant period [64.7% (95% CI 63.0–66.3) in Brazil and 82.6% (80.6–84.5) in Scotland], compared to the Delta dominant period [80.7% (77.8–83.3) in Brazil and 92.8% (85.7–96.4) in Scotland]. From 27 days onwards, VE started to decline in both countries. However, protection against severe COVID-19 (hospitalisation or death) remained high from 28 days after the second dose or at 98 days or more after the second dose [19].

A systematic review of 22 published studies and two ongoing trials evaluated VE against COVID-19 infection in healthy and immunosuppressed children and adolescents aged 2–21 years old [20]. This found that the immune response and efficacy in protecting against moderate to severe COVID-19 infection of COVID-19 vaccines were 96–100% in healthy children and adolescents. Specially, VE against COVID-19 related hospitalisation and its consequences after first and second doses were 91% (95% 89–92) and 92% (95% CI 76–100) respectively. VE was lower in those with underlying diseases and suppressed immune systems [20].

## VACCINE SAFETY

There is considerable health policy, public health and public interest in the safety of COVID-19 vaccines, not least because of their very rapid developmental timelines. This interest in safety signals has centred on vascular and neurological adverse events.

### Vaccination and risk of vascular complications

An analysis using the EAVE II platform found no positive associations between the first dose BNT162b2 and thrombocytopenic, thromboembolic and haemorrhagic events, but there was a small increase risk of idiopathic thrombocytopenic purpura (ITP) in those receiving a first dose of ChAdOx1 nCoV-19 [21^▪▪^]. Second dose ChAdOx1 nCoV-19 vaccination was also observed to be associated with borderline increased risks of ITP and cerebral venous sinus thrombosis (CVST) events [22]. This small elevated risk of CVST events following ChAdOx1 nCoV-19 vaccination was also observed based on a pooled self-controlled case series study of 3 UK nations undertaken using the DaC-VaP platform [23].

More recently, attention has focused on risk of myocarditis. Studies have reported that the risk of myocarditis is greater after SARS-CoV-2 infection than after sequential doses of BNT162b2 COVID-19 vaccination [24^▪▪^,^25^]; the incidence rate ratio (IRR) of myocarditis was 11.14 (95% CI 8.64–14.36) following a positive SARS-CoV-2 test before vaccination and 5.97 (95% CI 4.54–7.87) following a positive SARS-CoV-2 test after vaccination, compared to 1.52 (95% CI 1.24–1.85) after first dose of BNT162b2, 1.57 (95% CI 1.28–1.92) after second dose, 1.72 (95% CI 1.33–2.22) after a booster dose [24^▪▪^].

### Vaccination and risk of neurological complications

In an analysis across England with validation in Scotland, there was a small increased risk of neurological complications – in particular Guillain-Barré syndrome, Bell's palsy and haemorrhagic stroke in those who received COVID-19 vaccines, but the risk of these complications was greater following a positive SARS-CoV-2 test [26^▪▪^,^27^]. It was estimated that there were 38 excess cases of Guillain-Barré syndrome per 10 million people after receiving ChAdOx1 nCoV-19 and 145 excess cases after a positive SARS-CoV-2 test [26^▪▪^]. Overall, these adverse events were rare thus providing reassurance about the safety of COVID-19 vaccines.

## CONCLUSION

There is now a substantial body of evidence demonstrating that the three main COVID-19 vaccines deployed in the UK offer considerable protection against symptomatic COVID-19 infection and in particular against severe forms of the disease leading to COVID-19 related hospital admission and mortality. This VE has remained high as new variants have emerged, particularly in those who are fully vaccinated. Studies have found that VE is also high in important sub-populations, including pregnant women, children and young people and the elderly. Vaccine protection does however wane underscoring the need for vaccine boosters. The safety profile of COVID-19 vaccines has now also been extensively studied, these investigations finding small increases in risks of vascular and neurological events associated with some vaccines, but overall lower risks of these outcomes than following SARS-CoV-2 infection. There is a need to continue to investigate vaccine effectiveness and safety as we move into a new phase of the pandemic.

## Acknowledgements

*The present paper is part of the EAVE II collaboration's research output on COVID-19 pandemic. T.S., C.R. and A.S. conceptualised and drafted the manuscript. All authors provided important comments, contributed to the final manuscript and checked for important intellectual content.*

### Financial support and sponsorship

*EAVE II is funded by the Medical Research Council (MR/R008345/1) with the support of BREATHE–The Health Data Research Hub for Respiratory Health (MC_PC_19004), which is funded through the UK Research and Innovation Industrial Strategy Challenge Fund and is delivered through Health Data Research UK. Additional support has been provided through Public Health Scotland and Scottish Government Director- General Health and Social Care. We acknowledge the support of the EAVE II Patient Advisory Group.*

### Conflicts of interest

*A.S. and C.R. are members of the Scottish Government Chief Medical Officer's COVID-19 Advisory Group. A.S. is a member of the Scottish Government's Standing Committee on Pandemic Preparedness, the UK Government's New and Emerging Respiratory Virus Threats Advisory Group (known as NERVTAG) Risk Stratification Subgroup, the Department of Health and Social Care's COVID-19 Therapeutics Modelling Group, and was a member of AstraZeneca's COVID-19 Strategic Thrombocytopenia Taskforce. All A.S.'s roles are unfunded. C.R. is a member of the Scientific Pandemic Influenza Group on Modelling, Medicines and Healthcare products Regulatory Agency Vaccine Benefit and Risk Working Group. All other authors declare no competing interests.*

## REFERENCES AND RECOMMENDED READING

Papers of particular interest, published within the annual period of review, have been highlighted as:

▪ of special interest
▪▪ of outstanding interest
